# Modified ASPECTS for DWI including deep white matter lesions predicts subsequent intracranial hemorrhage

**DOI:** 10.1007/s00415-012-6446-1

**Published:** 2012-02-17

**Authors:** Hiroyuki Kawano, Teruyuki Hirano, Makoto Nakajima, Yuichiro Inatomi, Toshiro Yonehara, Makoto Uchino

**Affiliations:** 1 Department of Neurology, Faculty of Life Sciences, Kumamoto University, 1-1-1 Honjo, Kumamoto, 860-0811 Japan; 2 Department of Neurology, Stroke Center, Saiseikai Kumamoto Hospital, 5-3-1 Chikami, Kumamoto, 861-4193 Japan

**Keywords:** Thrombolysis, Diffusion-weighted imaging, Tissue plasminogen activator, Alberta Stroke Programme Early CT Score, Intracranial hemorrhage, White matter

## Abstract

We hypothesized that extensive early ischemic changes increase subsequent intracranial hemorrhage (ICH) in patients within 3 h of onset regardless of intravenous tPA (IV-tPA). We have established a modified scoring method, ASPECTS+W, including deep white matter lesions on DWI (DWI-W) in addition to the original ASPECTS regions. We aimed to elucidate whether CT-ASPECTS, DWI-ASPECTS, and ASPECTS+W could be useful tools in helping to predict subsequent ICH in acute ischemic stroke. One-hundred sixty-four consecutive patients with anterior circulation ischemic stroke were enrolled. All patients underwent both MRI and CT within 3 h of onset. ASPECTS+W was defined as an 11-point method combining the ten ASPECTS regions and DWI-W. The relationships of CT-ASPECTS, DWI-ASPECTS, and ASPECTS+W with ICH within the initial 36 h were assessed. Thirty-six patients (22%) were treated with IV-tPA. Follow-up CT was obtained in 159 patients, and 19 (12%) developed ICH. Patients with ICH had higher baseline NIHSS scores (median, 25 vs. 13, *p* = 0.010), a higher rate of IV-tPA (42 vs. 20%, *p* = 0.041), lower CT-ASPECTS (median, 7 vs. 10, *p* = 0.008), lower DWI-ASPECTS (6 vs. 9, *p* = 0.001), lower ASPECTS+W (6 vs. 9, *p* = 0.001), and higher DWI-W lesions (74 vs. 47%, *p* = 0.048) than those without ICH. ICA or M1 proximal occlusion was more frequently seen in patients with ICH (68 vs. 32%, *p* = 0.004) than in those without ICH. On multivariate regression analysis, lower ASPECTS+W (OR 0.75, 95% CI 0.58–0.96, *p* = 0.027) and administration of IV-tPA (OR 9.13, 95% CI 2.15–46.21, *p* = 0.004) independently predicted ICH development. In conclusion, ASPECTS+W is a useful tool for predicting ICH development independent of IV-tPA.

## Introduction

**Fig. 1:**
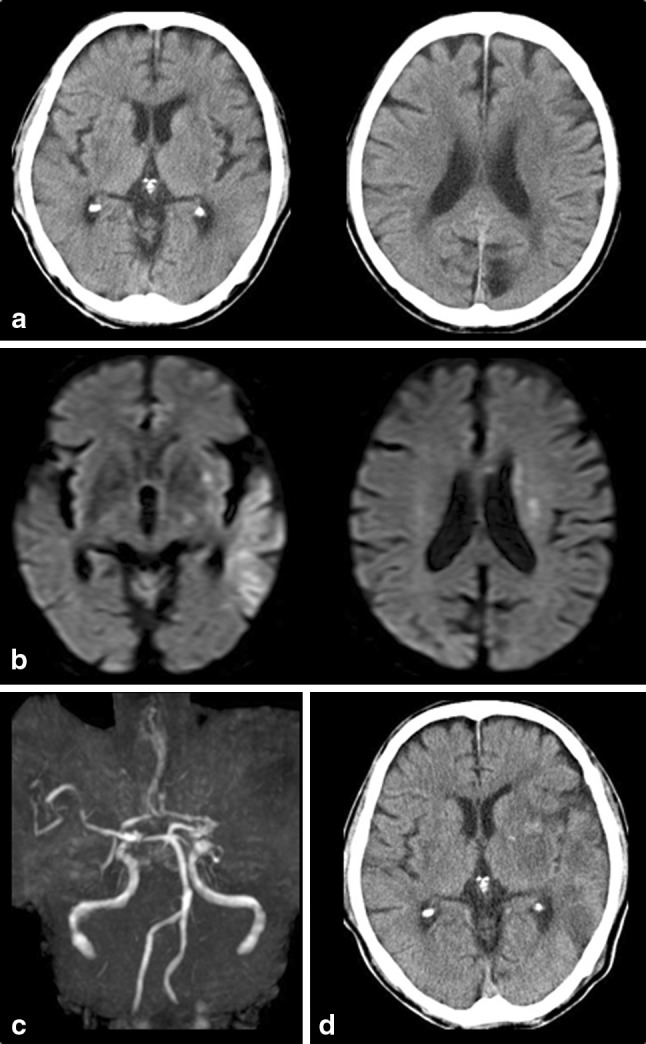
Case 1: 85-year-old man. Initial CT at 131 min of onset shows early ischemic change in the left L, I, and M3 (CT-ASPECTS, 7) (**a**). Initial DWI at 137 min of onset shows the hyperintense lesion in the left L, I, M2, M3, and W (DWI-ASPECTS, six and ASPECTS+W, six) (**b**). MRA on admission (**c**) shows occlusion of the left M1 proximal portion. The NIHSS score before IV-tPA was 19. IV-tPA was started 176 min of onset. The NIHSS score after 24 h was 15. The patient had asymptomatic ICH (HI 1) 24 h after tPA injection (**d**). The modified Rankin scale score at 90 days was three

Extensive early ischemic changes (EICs) on CT [1–5] or diffusion-weighted imaging (DWI) [6–9] have been identified as markers of an increased risk of thrombolysis-related intracranial hemorrhage (ICH). A quick and standardized CT scoring system, the Alberta Stroke Programme Early CT Score (ASPECTS) [10], has been increasingly applied to DWI images (DWI-ASPECTS) in several studies [9, 11, 12]. Its high sensitivity and good inter-rater agreement for the detection of EICs [13] make it feasible to use DWI-ASPECTS as a practical tool for selecting patients for thrombolysis (Fig. 1).

**Fig. 2:**
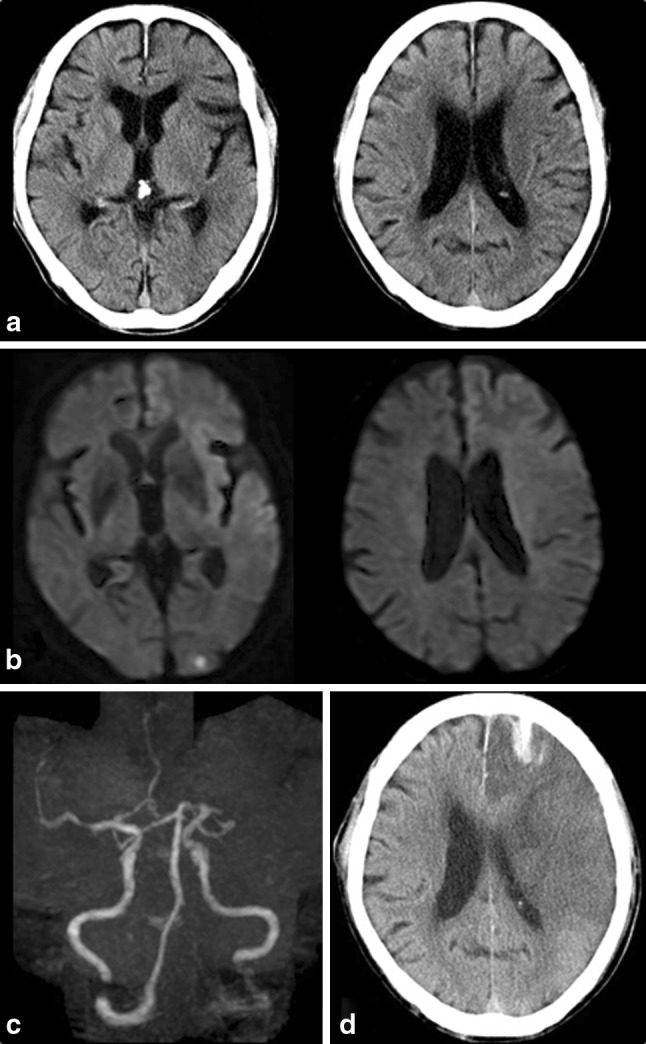
Case 2: 67-year-old man. Initial DWI at 92 min of onset shows the hyperintense lesion in the left I, M1, M2, M4, M5, and W (DWI-ASPECTS, five and ASPECTS+W, five) (**a**). Initial CT at 108 min of onset shows early ischemic change in the left L, I, M1, M2, and M3 (CT-ASPECTS, five) (**b**). MRA on admission (**c**) shows occlusion of the left M1 proximal portion. The NIHSS score on admission was 25. The patient was not treated with tPA, but had symptomatic ICH (PH1) 18 h of onset (**d**). The NIHSS score after 24 h was 38. The patient died on day four

Several studies have reported that it is possible to predict ICH risk using DWI-ASPECTS in patients treated by IV-tPA within 3 h [12] and by IV/IA thrombolysis within 6 h [9]. However, these results were based on thrombolysed patients only, and a substantial number of patients was excluded because of extensive EIC. Although lower DWI-ASPECTS was associated with increased ICH risk, patients with DWI-ASPECTS >7 still had a 3–4% ICH risk [9, 11, 12] (Fig. 2).

To determine the clinical utility of the ASPECTS system for ICH risk stratification, especially with respect to patient selection for IV-tPA within a 3-h time window, a controlled study will be required that includes all patients enrolled into active therapy regardless of their EIC size. However, such a study poses an ethical problem, because extensive EIC is established as a contraindication for thrombolysis in several guidelines from the United States [14], Canada [15], Europe [16], and Japan [17].

As an alternative approach to overcoming this issue, we designed a retrospective study including all patients with and without thrombolysis in whom both CT and DWI were performed within 3 h of onset. We hypothesized that extensive EIC increases subsequent ICH in patients within 3 h of onset regardless of IV-tPA. We have established a modified scoring method, ASPECTS+W, including DWI-W lesions, which has been found to be a negative predictor of early dramatic improvement after IV-tPA [18], in addition to each of the ten original ASPECTS regions. We aimed to elucidate whether CT-ASPECTS, DWI-ASPECTS, and ASPECTS+W could be useful tools to avoid subsequent ICH in patients with acute ischemic stroke.

## Subjects and methods

### Patients

Consecutive, hyperacute, ischemic stroke patients admitted to our stroke center within 3 h of symptom onset between October 2005 and June 2008 were registered. Of these, patients who underwent both CT and MRI within 3 h of onset were enrolled in the present study. In patients treated with IV-tPA, both CT and MRI were performed before the initiation of IV-tPA. Patients with pacemakers were excluded, because MRI is contraindicated in such patients. The relationship between the extent of EICs and the development of ICH within the initial 36 h of onset was assessed. Written, informed consent to treatment and participation in this study was obtained from all patients or their next of kin. The institutional ethics committee approved our research protocol.

The inclusion and exclusion criteria for IV-tPA used were in accordance with J-ACT [19]. Baseline CTs and DWIs were assessed for ischemic changes and quantified using ASPECTS. The presence of ischemic lesions was evaluated, and thrombolytic decision-making was done by the physician in charge. Stroke neurologists assessed National Institute of Health Stroke Scale (NIHSS) [20] scores on admission or before IV-tPA. Based on the neurological, radiological, cardiological, and hematological profiles, the stroke subtype was determined according to the Trial of Org 10172 in Acute Stroke Treatment (TOAST) classification system [21] by a consensus of stroke neurologists at our institute.

### CT and MR imaging

All CT scans were obtained using a multidetector CT scanner (Aquilion 16; Toshiba Medical Systems, Tochigi, Japan). The scanning parameters of the unenhanced CT were 120 kV and 500 mA, with a 512 × 512 image matrix, 24-cm field of view, and 8-mm section thickness. MR imaging was performed using a 1.5-T unit (Toshiba Medical Systems) with echo-planar imaging sequences, including diffusion. The typical stroke MRI protocol consisted of DWI and 3D time-of-flight MR angiography (MRA). Diffusion-weighted imaging was obtained with *b* values of 1,000 s/mm^2^.

CT-ASPECTS and DWI-ASPECTS were evaluated according to the ten ASPECTS regions method. For a better evaluation of ischemic extent, we have established a modified scoring method, ASPECTS+W, including DWI-W lesions [18] in addition to each of the ten original ASPECTS regions, assigning a score of 1 for a normal and a score of 0 for a region showing ischemic change. Briefly, we defined ASPECTS+W as an 11-point method. A DWI-W lesion was defined as a hyperintensity lesion in the corona radiata. The outermost limit of DWI-W was the subcortex of the M5 region, and the innermost limit was the caudate nucleus. Diffusion-weighted imaging-W lesions were evaluated at the level 2 cm superior to the thalamus and striatum [18]. Intracranial hemorrhage was defined as CT evidence of new hemorrhagic infarction (HI) or parenchymal hematoma (PH) within the initial 36 h [4]*.* Symptomatic ICH (sICH) was defined as an increase of one or more in the NIHSS score compared with that on admission. Arterial occlusion was assessed on the initial MRA.

For determining the ASPECTS values, CT and DWI were evaluated independently for each image at separate sessions and off line. Three stroke neurologists (H.K., T.H., and M.N.) independently and retrospectively evaluated CT-ASPECTS, DWI-ASPECTS, DWI-W lesions, and subsequent ICH. The imaging interpretations were performed blinded to clinical information and the outcome imaging findings. Disagreements were resolved by consensus.

### Statistical analysis

Kappa statistics were used to assess the investigators’ agreement about CT-ASPECTS, DWI-ASPECTS, and DWI-W lesions. The significance of intergroup differences was assessed using Fisher’s exact test for categorical variables and the Wilcoxon rank-sum test for continuous variables. To obtain the cut-off point of CT-ASPECTS, DWI-ASPECTS, and ASPECTS+W to predict ICH within the initial 36 h, receiver operating characteristic (ROC) curves were constructed. Multivariate analysis was performed to identify factors that independently predict development of ICH within the initial 36 h. Variables with a value of *p* < 0.1 on univariate analysis were included in the multivariate models. Lesion size was entered into the three models: CT-ASPECTS (Model 1), DWI-ASPECTS (Model 2), and ASPECTS+W (Model 3). Values of *p* < 0.05 were considered significant. Statistical analyses were performed using the JMP 7 package (SAS Institute Inc., Cary, NC, USA).

## Results

Overall, 791 consecutive acute ischemic stroke patients were admitted to our stroke center within 3 h of onset (cerebral infarction, 715 patients; transient ischemic attack, 76 patients); 627 were excluded because of posterior circulation stroke (*n* = 215), lack of CT or MRI study (*n* = 336) within 3 h of onset, or diagnosis of TIA (*n* = 76). As a result, 164 patients with hyperacute anterior circulation ischemic stroke who underwent both CT and MRI within 3 h of onset were enrolled (91 males, 73 females; mean age ± SD, 75.2 ± 11.7 years).

The median baseline NIHSS score of the 164 patients was 14 (range, 0–40). The baseline NIHSS score was higher in patients with cardioembolism (CE) than with other stroke subtypes (median, 21.5 vs. 7.5, *p* < 0.001). The median (range) baseline CT-ASPECTS, DWI-ASPECTS, and ASPECTS+W values were 10 (0–10), 8 (0–10), and 9 (0–11), respectively. The kappa statistics of agreement among the three investigators for CT-ASPECTS and DWI-ASPECTS were 0.344 and 0.434, respectively. Diffusion-weighted imaging-W lesions were found in 82 patients (50%). The kappa value for the presence of DWI-W lesions was 0.605.

Of the 164 patients, 36 (22%) were treated with IV-tPA. The mean time of onset to tPA bolus was 125.0 ± 29.5 min (range, 80–178 min). The time from entering the hospital to tPA bolus was 58.4 ± 15.8 min (range, 37–108 min). The median (range) baseline NIHSS score in patients with IV-tPA was 14 (7–31). The median (range) CT-ASPECTS, DWI-ASPECTS, and ASPECTS+W values were 9.5 (3–10), 8.5 (6–10), and 9 (6–11), respectively.

Of the 159 patients who had a follow-up CT study within the initial 36 h, 19 (12%) demonstrated ICH development (HI, 12 patients; PH, seven patients). Patients with ICH had higher NIHSS scores on admission (median, 23 vs. 13, *p* = 0.010), a higher rate of IV-tPA (42 vs. 20%, *p* = 0.041), lower CT-ASPECTS (median, 7 vs. 10, *p* = 0.008), lower DWI-ASPECTS (median, 6 vs. 9, *p* = 0.001), lower ASPECTS+W (median, 6 vs. 9, *p* = 0.001), and more frequent DWI-W lesions (74 vs. 47%, *p* = 0.048) than those without ICH. The presence of ICA or M1 proximal occlusion was more frequently seen in patients with than without ICH (68 vs. 32%, *p* = 0.004) (Table 1). On multivariate regression analysis, although CT-ASPECTS and DWI-ASPECTS did not predict development of ICH (Table 2, Model 1 and Model 2), lower ASPECTS+W (OR 0.75, 95% CI 0.58–0.96, *p* = 0.027) and administration of IV-tPA (OR 9.13, 95% CI 2.15–46.21, *p* = 0.005) were independently related to ICH development within the initial 36 h (Table 2, Model 3).

**Table 1:** Univariate statistical analysis comparing patients with and without development of intracranial hemorrhage (ICH)

	With ICH (*n* = 19)	Without ICH (*n* = 140)	*p* value
Age (mean ± SD years)	80.1 ± 9.2	74.8 ± 11.8	0.060
Sex (male)	11 (58%)	77 (55%)	0.664
IV-tPA	8 (42%)	28 (20%)	0.041
Hypertension	9 (47%)	100 (71%)	0.062
Diabetes mellitus	5 (26%)	34 (24%)	0.784
Dyslipidemia	3 (16%)	22 (16%)	1.000
Atrial fibrillation	12 (63%)	62 (44%)	0.145
Previous ischemic heart disease	2 (11%)	13 (9%)	0.695
Previous cerebrovascular disease	3 (16%)	34 (24%)	0.567
Antiplatelet agent use	3 (16%)	29 (21%)	0.767
Anticoagulant use	2 (11%)	26 (19%)	0.531
Platelet count on admission (×10^4^/μl)	18.7 ± 5.3	19.6 ± 5.3	0.522
Systolic blood pressure on admission (mmHg)	156.4 ± 23.4	161.3 ± 33.2	0.532
Diastolic blood pressure on admission (mmHg)	77.1 ± 20.0	86.8 ± 21.1	0.058
Glucose on admission (mg/dl)	144.6 ± 68.2	138.2 ± 51.3	0.626
NIHSS score on admission, median (IQR)	23(15–25)	12.5 (5–25)	0.010
CT-ASPECTS, median (IQR)	7 (5–10)	10 (7–10)	0.008
DWI-ASPECTS, median (IQR)	6 (3–8)	9 (6–10)	0.001
ASPECTS+W, median (IQR)	6 (3–8)	9 (6–10)	0.001
DWI-W lesions	14 (74%)	66 (47%)	0.048
Stroke subtype (TOAST classification)			0.355
Cardioembolism	14 (74%)	77 (55%)	
Large artery atherosclerosis	4 (21%)	26 (19%)	
Small vessel occlusion	0	15 (11%)	
Other causes	0	1 (15)	
Undetermined	1 (5%)	21 (15%)	
Presence of ICA/M1 proximal occlusion	13(68%)	44(32%)	0.004

*IQR* interquartile range *ICH* both symptomatic and asymptomatic ICH

**Table 2:** Multivariate analysis for development of intracranial hemorrhage

	OR	95% CI	*p* value
Model 1 (CT-ASPECTS)			
Age (per year)	1.05	0.98–1.13	0.180
Sex (female)	0.53	0.15–1.68	0.292
IV–tPA	6.01	1.58–25.60	0.010
NIHSS score on admission (per point increase)	1.02	0.95–1.10	0.549
Presence of hypertension	2.28	0.75–7.16	0.147
Diastolic blood pressure on admission (per mmHg increase)	0.98	0.95–1.01	0.143
CT-ASPECTS (per point increase)	0.82	0.64–1.03	0.094
Presence of ICA/M1 proximal occlusion	2.74	0.80–10.33	0.118
Model 2 (DWI-ASPECTS)			
Age (per year)	1.06	0.99–1.14	0.113
Sex (female)	0.53	0.15–1.71	0.300
IV-tPA	8.88	2.01–46.49	0.005
NIHSS score on admission (per point increase)	1.00	0.92–1.09	0.942
Presence of hypertension	2.11	0.68–6.73	0.198
Diastolic blood pressure on admission (per mmHg increase)	0.98	0.95–1.01	0.222
Presence of ICA/M1 proximal occlusion	1.96	0.53–7.72	0.316
DWI-ASPECTS (per point increase)	0.76	0.57–1.00	0.056
Presence of DWI-W lesion	0.68	0.17–2.44	0.555
Model 3 (ASPECTS+W)			
Age (per year)	1.06	0.99–1.14	0.107
Sex (female)	0.53	0.15–1.71	0.302
IV-tPA	9.13	2.15–46.21	0.004
NIHSS score on admission (per point increase)	1.00	0.92–1.09	0.949
Presence of hypertension	2.09	0.67–6.63	0.201
Diastolic blood pressure on admission (per mmHg increase)	0.98	0.95–1.01	0.225
ASPECTS+W (per point increase)	0.75	0.58–0.96	0.027
Presence of ICA/M1 proximal occlusion	1.97	0.54–7.75	0.313

*OR* odds ratio, *CI* confidence interval

The optimal cut-off point of ASPECTS+W to predict development of ICH within the initial 36 h was <8, with a sensitivity of 79%, specificity of 60%, and an area under the ROC curve of 0.732 (*p* = 0.002). Four patients (3%) had sICH; no patients were treated with IV-tPA because of extensive ischemic lesions, all had a poor outcome (modified Rankin Scale score 5 or 6), and three patients had DWI-W lesions. The values of DWI-ASPECTS and ASPECTS+W tended to be lower in patients with than without sICH (median, 5 vs. 8, *p* = 0.131, and 5 vs. 9, *p* = 0.066), but the values on CT were not significantly different (median 5 vs. 8, *p* = 0.592).

## Discussion

In the present study, the relationships of baseline DWI-ASPECTS and CT-ASPECTS with early development of ICH were assessed in patients with hyperacute ischemic stroke involving the anterior circulation who received IV-tPA and in those who did not. A low ASPECTS+W value, which is based on a modified 11-point method, and administration of IV-tPA were independently related to development of ICH within the initial 36 h, although CT-ASPECTS and DWI-ASPECTS were not significant predictors of ICH development. Thus, patients with baseline ASPECTS+W values of less than eight points are at high risk for ICH development in the acute phase.

There are some previous studies that examined whether CT-ASPECTS or DWI-ASPECTS could predict ICH development. On the other hand, there is controversy about whether CT-ASPECTS can help predict thrombolysis-related ICH [22, 23]*.* In ECASS II, for acute ischemic stroke patients within 6 h of onset, low CT-ASPECTS values were related to an increased risk for thrombolysis-related ICH [23]. In the NINDS rt-PA Stroke Study population, for patients within 3 h of onset, baseline CT-ASPECTS was not significantly related to thrombolysis-related ICH [22]. It was reported that DWI-ASPECTS predicted symptomatic ICH after IV/IA thrombolysis within 6 h of onset [9]. The present study was designed in several different ways: it included patients treated with and without IV-tPA, within 3 h of onset, examined by both CT and MRI including DWI and MRA, and it was a non-randomized, controlled study. In these differences, the present study is the first-ever report comparing ASPECTS values on both CT and DWI in hyperacute ischemic stroke patient regardless of IV-tPA within 3 h of onset.

The present study is unique in that DWI-W lesions were evaluated in addition to the ten ASPECTS regions, and the ASPECTS+W method was proposed. We have previously shown that absence of DWI-W lesions was an independent predictor of early dramatic improvement after IV-tPA [18]. Detection of EICs in deep white matter is difficult on CT. Although patients with ICH had DWI-W lesions more frequently than those without ICH on univariate analysis, DWI-W itself was not significantly related to ICH on multivariate regression analysis. The reason for this might be that patients with only DWI-W lesions, who were diagnosed as having small vessel occlusion, were included. However, using the 11-point method, rather than the original ten-point method, ASPECTS+W had a good correlation with ICH development within the initial 36 h. We believe that evaluation of DWI-W lesions in addition to the 10 DWI-ASPECTS regions is a more useful method than CT-ASPECTS and DWI-ASPECTS for predicting which patients are prone to ICH.

Low ASPECTS+W values were related to ICH development regardless of IV-tPA. Because time is limited before IV-tPA and to avoid development of ICH, patients with somewhat extensive ischemic lesions might be excluded by the physician in charge. In the present study, we tried to elucidate which ASPECTS method is the most helpful for avoiding hemorrhagic complications. Although this was a non-randomized, controlled study, we think that we could decrease the potential bias by using CT and DWI within 3 h of symptom onset and by including all patients eligible for IV-tPA according to the time window. If we were to administer IV-tPA to patients with low ASPECTS+W values, the risk of thrombolysis-related ICH might be increased. In the present study, the optimal cut-off point of ASPECTS+W to predict ICH was <8 points. This result suggests that patients with ASPECTS+W values ≥ 8 points could be safely-administered IV-tPA.

White matter is less metabolically active than gray matter and requires a reduced blood flow per volume of tissue [24]. Additionally, whether the deep white matter progresses to an infarction depends on the collateral blood supply. Therefore, the presence of a DWI-W lesion might indicate the presence of severe ischemia. The fundamental mechanism of hemorrhagic transformation is disruption of the permeability of the blood–brain barrier through ischemia [25]. Although recanalization of the occluded artery was not evaluated in the present study, the ASPECTS+W method might be associated with the severity of ischemia and predicts ICH development.

In the present study, CT-ASPECTS could not predict ICH independently of IV-tPA. This result was similar to that found in the NINDS rt-PA Stroke Study population [22], and different from that in ECASSII [23]. Because patients within 6 h of onset were included in ECASS II, time might be a major factor in the difference. Although the utility of CT cannot be denied, CT was not sufficient to predict hemorrhagic complications in the hyperacute phase.

The present study had some limitations. First, the decision to administer IV-tPA was at the discretion of the attending stroke neurologists. Therefore, low CT-ASPECTS, DWI-ASPECTS, or ASPECTS+W values prohibited the use of IV-tPA, resulting in a skewed distribution of ASPECTS for patients treated with IV-tPA. Second, whether to have the patient undergo both DWI and CT within 3 h of onset was decided by the physician in charge. Third, the kappa values for CT-ASPECTS and DWI-ASPECTS were relatively low. There appear to have been some differences in whether the examiner assessed the lesion as positive or negative when there were slight ischemic changes or quite small lesions. Fourth, the present study was a non-randomized and retrospective study. Fifth, because sICH was quite infrequent (3%), whether ASPECTS+W can predict sICH is uncertain and warrants further study. Symptomatic worsening in patients with sICH appeared to be mainly due to the initial extensive ischemic lesions and brain edema. Sixth, MRI was performed after CT in most patients. When the physician in charge might decide that patients with extensive EICs on the initial CT were not indication for IV-tPA, MRI was not performed within 3 h of onset. Therefore, patients with extensive EICs on the initial CT might be excluded from the present study. Finally, because the present study is non-randomized, retrospective series, we think it is too early to conclude that we should withhold IV-tPA in patients with low ASPECTS+W.

In conclusion, the present study demonstrated that the ASPECTS+W method is a useful tool for predicting ICH independently from IV-tPA. Pre-treatment DWI provides useful information for predicting ICH development, but randomized trial is required to confirm whether ASPECTS+W method could be used as surrogates for treatment decision-making.

## References

[1] Larrue V, von Kummer R, del Zoppo G, Bluhmki E (1997). Hemorrhagic transformation in acute ischemic stroke. Potential contributing factors in the European Cooperative Acute Stroke Study. Stroke.

[2] Larrue V, von Kummer RR, Müller A, Bluhmki E (2001). Risk factors for severe hemorrhagic transformation in ischemic stroke patients treated with recombinant tissue plasminogen activator. A secondary analysis of the European–Australasian Acute Stroke Study (ECASS II). Stroke.

[3] Hacke W, Kaste M, Fieschi C, von Kummer R, Davalos A, Meier D, Larrue V, Bluhmki E, Davis S, Donnan G, Schneider D, Diez-Tejedor E, Trouillas P (1998). Randomised double-blind placebo-controlled trial of thrombolytic therapy with intravenous alteplase in acute ischaemic stroke (ECASS II). Lancet.

[4] Hacke W, Kaste M, Fieschi C, Toni D, Lesaffre E, von Kummer R, Boysen G, Bluhmki E, Höxter G, Mahagne MH, Hennerici M, ECASS Study Group (1995). Intravenous thrombolysis with recombinant tissue plasminogen activator for acute hemispheric stroke. JAMA.

[5] Cocho D, Borrell M, Martí-Fàbregas J, Montaner J, Castellanos M, Bravo Y, Molina-Porcel L, Belvís R, Díaz-Manera JA, Martínez-Domeño A, Martínez-Lage M, Millán M, Fontcuberta J, Martí-Vilalta JL (2006). Pretreatment hemostatic markers of symptomatic intracerebral hemorrhage in patients treated with tissue plasminogen activator. Stroke.

[6] Lansberg MG, Thijs VN, Bammer R, Kemp S, Wijman CA, Marks MP, Albers GW, DEFUSE Investigators (2007). Risk factors of symptomatic intracerebral hemorrhage after tPA therapy for acute stroke. Stroke.

[7] Singer OC, Humpich MC, Fiehler J, Albers GW, Lansberg MG, Kastrup A, Rovira A, Liebeskind DS, Gass A, Rosso C, Derex L, Kim JS, Neumann-Haefelin T, MR Stroke Study Group Investigators (2008). Risk for symptomatic intracerebral hemorrhage after thrombolysis assessed by diffusion-weighted magnetic resonance imaging. Ann Neurol.

[8] Singer OC, Berkefeld J, Lorenz MW, Fiehler J, Albers GW, Lansberg MG, Kastrup A, Rovira A, Liebeskind DS, Gass A, Rosso C, Derex L, Kim JS, Neumann-Haefelin T, MR Stroke Study Group Investigators (2009). Risk of symptomatic intracerebral hemorrhage in patients treated with intra-arterial thrombolysis. Cerebrovasc Dis.

[9] Singer OC, Kurre W, Humpich MC, Lorenz MW, Kastrup A, Liebeskind DS, Thomalla G, Fiehler J, Berkefeld J, Neumann-Haefelin T, MR Stroke Study Group Investigators (2009). Risk assessment of symptomatic intracerebral hemorrhage after thrombolysis using DWI-ASPECTS. Stroke.

[10] Barber PA, Demchuk AM, Zhang J, Buchan AM, ASPECTS Study Group. Alberta Stroke Programme Early CT Score (2000). Validity and reliability of a quantitative computed tomography score in predicting outcome of hyperacute stroke before thrombolytic therapy. Lancet.

[11] Kimura K, Iguchi Y, Shibazaki K, Terasawa Y, Inoue T, Uemura J, Aoki J (2008). Large ischemic lesions on diffusion-weighted imaging done before intravenous tissue plasminogen activator thrombolysis predicts a poor outcome in patients with acute stroke. Stroke.

[12] Nezu T, Koga M, Kimura K, Shiokawa Y, Nakagawara J, Furui E, Yamagami H, Okada Y, Hasegawa Y, Kario K, Okuda S, Nishiyama K, Naganuma M, Minematsu K, Toyoda K (2010). Pretreatment ASPECTS on DWI predicts 3-month outcome following rt-PA: SAMURAI rt-PA Registry. Neurology.

[13] Fiebach JB, Schellinger PD, Jansen O, Meyer M, Wilde P, Bender J, Schramm P, Jüttler E, Oehler J, Hartmann M, Hähnel S, Knauth M, Hacke W, Sartor K (2002). CT and diffusion-weighted MR imaging in randomized order: diffusion-weighted imaging results in higher accuracy and lower interrater variability in the diagnosis of hyperacute ischemic stroke. Stroke.

[14] Adams HP Jr, del Zoppo G, Alberts MJ, Bhatt DL, Brass L, Furlan A, Grubb RL, Higashida RT, Jauch EC, Kidwell C, Lyden PD, Morgenstern LB, Qureshi AI, Rosenwasser RH, Scott PA, Wijdicks EF; American Heart Association; American Stroke Association Stroke Council; Clinical Cardiology Council; Cardiovascular Radiology and Intervention Council; Atherosclerotic Peripheral Vascular Disease and Quality of Care Outcomes in Research Interdisciplinary Working Groups (2007) Guidelines for the early management of adults with ischemic stroke: a guideline from the American Heart Association/American Stroke Association Stroke Council, Clinical Cardiology Council, Cardiovascular Radiology and Intervention Council, and the Atherosclerotic Peripheral Vascular Disease and Quality of Care Outcomes in Research Interdisciplinary Working Groups: the American Academy of Neurology affirms the value of this guideline as an educational tool for neurologists. Stroke 38:1655–1711 10.1161/STROKEAHA.107.181486 17431204

[15] Norris JW, Buchan A, Cote R, Hachinski V, Phillips SJ, Shuaib A, Silver F, Simard D, Teal P (1998). Canadian guidelines for intravenous thrombolytic treatment in acute stroke. A consensus statement of the Canadian Stroke Consortium. Can J Neurol Sci.

[16] European Stroke Organisation (ESO) Executive Committee; ESO Writing Committee (2008). Guidelines for management of ischaemic stroke and transient ischaemic attack 2008. Cerebrovasc Dis.

[17] Shinohara Y, Yamaguchi T (2008). Outline of the Japanese Guidelines for the Management of Stroke 2004 and subsequent revision. Int J Stroke.

[18] Kawano H, Hirano T, Inatomi Y, Terasaki T, Yonehara T, Uchino M (2010). Presence of deep white matter lesions on diffusion-weighted imaging is a negative predictor of early dramatic improvement after intravenous tissue plasminogen activator thrombolysis. Cerebrovasc Dis.

[19] Yamaguchi T, Mori E, Minematsu K, Nakagawara J, Hashi K, Saito I, Shinohara Y, Japan Alteplase Clinical Trial (J-ACT) Group (2006). Alteplase at 0.6 mg/kg for acute ischemic stroke within 3 hours of onset: Japan Alteplase Clinical Trial (J-ACT). Stroke.

[20] Lyden P, Brott T, Tilley B, Welch KM, Mascha EJ, Levine S, Haley EC, Grotta J, Marler J, NINDS TPA Stroke Study Group (1994). Improved reliability of the NIH Stroke Scale using video training. Stroke.

[21] Adams HP, Bendixen BH, Kappelle LJ, Biller J, Love BB, Gordon DL, Marsh EE (1993). Classification of subtype of acute ischemic stroke. Definitions for use in a multicenter clinical trial. TOAST. Trial of Org 10172 in Acute Stroke Treatment. Stroke.

[22] Demchuk AM, Hill MD, Barber PA, Silver B, Patel SC, Levine SR, NINDS rtPA Stroke Study Group, NIH (2005). Importance of early ischemic computed tomography changes using ASPECTS in NINDS rtPA Stroke Study. Stroke.

[23] Dzialowski I, Hill MD, Coutts SB, Demchuk AM, Kent DM, Wunderlich O, von Kummer R (2006). Extent of early ischemic changes on computed tomography (CT) before thrombolysis: prognostic value of the Alberta Stroke Program Early CT Score in ECASS II. Stroke.

[24] Ransom BR, Acharya AB, Goldberg MP (2004) Molecular pathophysiology of white matter anoxic-ischemic injury. In: Mohr JP, Choi DW, Grotta JC, Wier B, Wolf PA (eds) Stroke. Pathophysiology, Diagnosis, and Management, 4th edn. Churchill Livingstone, New York, pp 867–881

[25] Khatri P, Wechsler LR, Broderick JP (2007). Intracranial hemorrhage associated with revascularization therapies. Stroke.

